# The Cytoprotective Effect of Hyperoside against Oxidative Stress Is Mediated by the Nrf2-ARE Signaling Pathway through GSK-3β Inactivation

**DOI:** 10.1371/journal.pone.0145183

**Published:** 2015-12-16

**Authors:** Hai-Yan Xing, Yong-Qing Cai, Xian-Feng Wang, Lin-Li Wang, Pan Li, Guan-Ying Wang, Jian-Hong Chen

**Affiliations:** Department of Pharmacy, Daping Hospital & Research Institute of Surgery, Third Military Medical University, Chongqing 400042, China; Emory University, UNITED STATES

## Abstract

Glycogen synthase kinase-3β (GSK-3β) acts as a negative regulator of NF-E2 related factor 2 (Nrf2) by inducing Nrf2 degradation and nuclear export. Our previous study demonstrated that the flavonoid hyperoside elicits cytoprotection against oxidative stress by activating the Keap1-Nrf2-ARE signaling pathway, thus increasing the expression of antioxidant enzymes, such as heme oxygenase-1 (HO-1), superoxide dismutase (SOD) and catalase. However, the role of GSK-3β in hyperoside-mediated Nrf2 activation is unclear. Here, we demonstrate that in a normal human hepatocyte cell line, (L02), hyperoside is capable of inducing the phosphorylation of GSK-3β at Ser9 without affecting the protein levels of GSK-3β and its phosphorylation at Thr390. Lithium chloride (LiCl) and short interfering RNA (siRNA)-mediated inhibition of GSK-3β significantly enhanced the ability of hyperoside to protect L02 liver cells from H_2_O_2_-induced oxidative damage, leading to increased cell survival shown by the maintenance of cell membrane integrity and elevated levels of glutathione (GSH), one of the endogenous antioxidant biomarkers. Further study showed that LiCl and siRNA-mediated inhibition of GSK-3β increased hyperoside-induced HO-1 expression, and the effect was dependent upon enhanced Nrf2 nuclear translocation and gene expression. These activities were followed by ARE-mediated transcriptional activation in the presence of hyperoside, which was abolished by the transfection of the cells with Nrf2 siRNA. Furthermore, the siRNA-mediated inhibition of Keap1 also enhanced hyperoside-induced Nrf2 nuclear accumulation and HO-1 expression, which was relatively smaller than the effects obtained from GSK-3β siRNA administration. Moreover, Keap1 siRNA administration alone had no significant effect on the phosphorylation and protein expression of GSK-3β. Collectively, our data provide evidence that hyperoside attenuates H_2_O_2_ -induced L02 cell damage by activating the Nrf2-ARE signaling pathway through both an increase in GSK-3β inhibitory phosphorylation at Ser9 and an inhibition of Keap1 and that hyperoside-mediated GSK-3β inhibition exhibits more significant effects.

## Introduction

**Fig 1:**
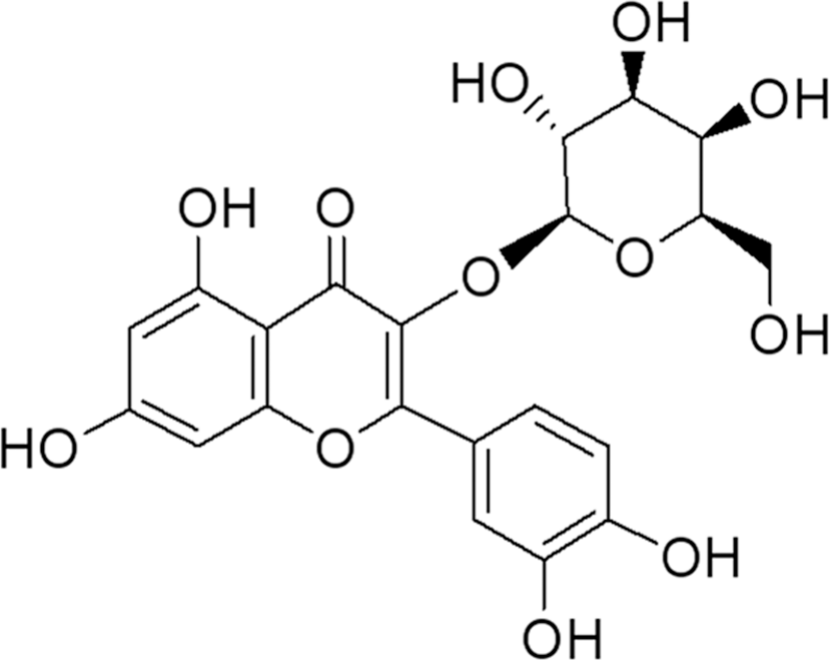
Chemical structure of hyperoside.

Hyperoside (Hyp, structure shown in **Fig 1**), a naturally occurring flavonoid present in fruits and vegetables, is a new promising agent in many models of disease prevention [1, 2]. Most of the protective effects of hyperoside against diseases are attributed to its antioxidative property [3, 4]. In addition to direct free radical scavenging and metal-chelation, hyperoside regulates the endogenous antioxidant defense system.

Studies have demonstrated that hyperoside protects against stimuli or ischemia/reperfusion-induced damage by increasing the activity of glutathione peroxidase (GSH-Px), catalase (CAT) [5] and superoxide dismutase (SOD) [3], as well as the expression of heme oxygenase-1 (HO-1) [6, 7]. The proposed mechanisms of hyperoside’s action involve enhancing the Kelch-like ECH-associated protein 1 (Keap1)-nuclear factor erythroid 2-related factor 2 (Nrf2)-antioxidant response element (ARE) signaling pathway. However, how hyperoside triggers Nrf2 activation and attenuates oxidative damage remains unclear.

The Keap1-Nrf2-ARE signaling axis serves as a "master regulator" in response to oxidative/electrophilic stresses and chemical insults through the induction of a large pool of cytoprotective genes [8]. Therefore, the activation of Nrf2 is considered as an important approach to preventing diseases triggered by stress and toxins, including liver diseases [9, 10]. Depending on the cellular redox balance, the Keap1-Nrf2-ARE signaling system is subjected to multiple layers of regulation [8]. Among these layers of regulation, the Keap1 or glycogen synthase kinase 3 (GSK-3)-mediated Nrf2 degradation and nuclear export is of particular importance. Under basal conditions, Keap1 acts as an adaptor between Nrf2 and the ubiquitination ligase Cullin-3 (Cul3) and promotes the proteasomal degradation of Nrf2. Upon modification of specific thiols by insult, Keap1 allows Nrf2 to translocate into the nucleus to promote the expression of a wide array of cytoprotective genes by binding to ARE in regulatory regions [11]. In addition to being regulated by the ubiquitin E3 ligase adapter Keap1, recent studies have identified GSK-3β as a novel regulator of Nrf2. GSK-3β phosphorylates a group of Ser residues in the Neh6 domain of Nrf2 that overlap with an SCF/beta-TrCP destruction motif (DSGIS, residues 334 to 338) to promote Keap1-independent degradation [12]. Moreover, GSK-3β acts upstream of Fyn kinase, which phosphorylates tyrosine 568 of Nrf2, leading to the nuclear export of Nrf2 [13]. Nuclear accumulation of Nrf2 might be induced by the increased inhibitory phosphorylation of GSK-3β (Ser9 and Thr390) [14, 15].

Among Nrf2 inducers, flavonoids have been demonstrated as regulators of the GSK-3β-associated signaling pathway in Nrf2 activation. Puerarin, one of the most extensively studied flavonoids, induces nuclear translocation of Nrf2 and stimulates the expression of Nrf2-dependent genes in APP/PS1 transgenic mice and neuron cells through the activation of the phosphatidylinositol 3-kinase (PI3K)/GSK-3β pathway [16–18]. Quercetin, the aglycone of hyperoside, improves hippocampus-dependent learning and memory in mice through the PI3K/protein kinase B (AKT)/Nrf2 pathway [19]. Flavonoid-mediated Nrf2 nuclear translocation might be mediated via the phosphorylation of mitogen-activated protein kinase (MAPK), AKT and GSK-3β in isolation or in concert [19–22]. However, the precise role of GSK-3β in the hyperoside-mediated nuclear translocation of Nrf2 and the expression of antioxidant genes remains to be elucidated.

The aim of this study was to investigate the capacity of hyperoside to inhibit hydrogen peroxide (H_2_O_2_)-induced oxidative stress in a normal human hepatocyte cell line, L02, by up-regulating the Nrf2-ARE signaling pathway through inactivation of GSK-3β. Our study demonstrates that hyperoside activates Nrf2 nuclear translocation and HO-1 expression through both GSK-3β inactivation and Keap1 inhibition, and that GSK-3β might act as a major integrator of multiple signaling cascades. These findings provide a novel insight into the antioxidant activity of hyperoside and its potential applications for the treatment of oxidative stress-related diseases.

## Materials and Methods

### Chemicals and reagents

Hyperoside (purity≥98%) was purchased from Nanjing Zelang Medical Technological Co., Ltd (Nanjing, Jiangsu, China). tert-butylhydroquinone (t-BHQ), sulforaphane (SFN), lithium chloride (LiCl) and H_2_O_2_ were purchased from Sigma Chemical (St. Louis, MO, USA). Mouse antibodies against Nrf2 (ab89443) were obtained from Abcam (Cambridge, UK). Rabbit antibodies against GSK-3β (3D10) (#9315), Keap1 (D6B12) (#8047) and Histone H2 (#2595) were purchased from Cell Signaling Technology (Beverly, MA, USA). Rabbit antibodies against HO-1 (SC-10789), mouse antibodies against GAPDH (SC-365062) and all secondary antibodies were obtained from Santa Cruz Biotechnology (Santa Cruz, CA, USA). All other chemicals were reagent-grade and purchased from Sigma-Aldrich, unless otherwise indicated.

### Cell culture

Normal human hepatocytes (L02 cells) obtained from Cell Bank of Type Culture Collection of the Chinese Academy of Sciences (Shanghai, China) were maintained in HyClone RPMI 1640 medium (Thermo Scientific, Beijing, China) supplemented with 10% (v/v) fetal bovine serum (Gibco, USA), 100 U/mL penicillin, and 100 μg/mL streptomycin at 37°C in a humidified atmosphere at 5% CO_2_. A hyperoside stock solution was prepared in dimethylsulfoxide (DMSO) and diluted with culture medium immediately prior to the experiment. Control cells were treated with equal amount of only DMSO at a final concentration of <0.1% (v/v). The dosage of hyperoside and LiCl (a specific inhibitor of GSK-3β) was based on published information [6, 23] and tested in pilot studies to ensure Nrf2 induction and GSK-3β inhibition, respectively. Oxidative stress conditions were produced by the addition of H_2_O_2_ to L02 cells.

### Cell viability and LDH leakage assays

The number of viable cells was counted using the Cell Counting Kit-8 colorimetric assay (Dojindo Laboratories, Kumamoto, Japan) according to the manufacturer’s instructions. Briefly, cells were grown on 96-well plates at a density of 5×10^3^ for 24 h. After treatment with 100 μM hyperoside and/or 100 μM H_2_O_2_ for the indicated time, the cells were incubated with 10 μl of the Cell Counting Kit-8 solution. After incubation at 37°C for 2 h in a humidified CO_2_ incubator, the absorbance was monitored at 450 nm on a microplate reader (Thermo Scientific, Waltham, MA, USA). The values obtained were normalized to those of control cells incubated with only the vehicle. The release of lactate dehydrogenase (LDH) was evaluated by the LDH assay kit (Genmed Scientifics, Wilmington, DE, USA), as previously described [24]. The obtained values were normalized to those of the maximum LDH released from completely lysed control cells.

### Glutathione quantification

The total glutathione (GSH) levels were determined by using the components provided in a glutathione assay kit obtained from Dojindo Laboratories (Kumamoto, Japan) according to the manufacturer's instructions. The total GSH levels were calculated from absorbance (405 nm) values on the basis of a calibration curve and normalized to the control group.

### Immunocytochemistry

Immunocytochemistry analysis was carried out according to the method described by Li et al. [25]. L02 cells grown on poly-L-lysine-coated coverslips were treated with hyperoside (100 μM) or t-BHQ (50 μM) for 6 h; then, the cells were washed with PBS, fixed in 4% paraformaldehyde for 30 min, permeabilized with 0.1% Triton-X and blocked in 10% non-fat dry milk in blocking buffer at 4°C for 12 h. Primary mouse anti-Nrf2 monoclonal antibody was added (1:400 dilution), and the cells were incubated overnight at 4°C. The cells were then incubated with the secondary antibody, Alexa Fluor® 488 goat anti-mouseIgG (H+L), at a 1:200 dilution for 1 h. After three further washes in PBS, the cells were counterstained with 1 μg/mL of 4,6-diamino-2-phenyl indole (DAPI) for 5 min. After washing with PBS, the cell slides were treated with a single drop of fluorescent mounting medium (Biomeda, Foster City, CA, USA) and observed under an inverted fluorescence microscope (Eclipse 80i; Nikon, Tokyo, Japan) at 400 x magnification.

### Short interfering RNA (siRNA) and transfection

Nrf2 siRNA, GSK-3β siRNA, Keap1 siRNA and control siRNAs designed and synthesized by Shanghai Gene Pharma Co., Ltd (Shanghai, China) were separately dissolved in Opti-MEM (Invitrogen, CA, USA). After 10 min of equilibration at room temperature, each siRNA solution was combined with the respective volume of the Lipofectamine 2000 solution (Invitrogen, CA, USA), mixed gently and allowed to form siRNA liposomes for 20 min. L02 cells were transfected with the transfection mixture in antibiotic-free cell culture medium for 48 h before hyperoside or t-BHQ treatment, and subjected to various measurements.

### Real-time RT-PCR

Total RNA was extracted from L02 cells using Trizol reagent (Invitrogen, Carlsbad, CA, USA). Equal amounts (2.0 μg) of total RNA were reverse-transcribed to cDNA by using the Transcriptor First Strand cDNA Synthesis Kit (Roche, Indianapolis, IN, USA) for use in PCR. The following primers were used for all of the PCR experiments: HO-1, TTTGAGGAGTTGCAGGAGC (forward) and AGGACCCATCGGAGAAGC (reverse); Nrf2, GCGCAGCTCCTACACCAACGC (forward) and CTGACGCAAGAAGCTCGCGGT (reverse); and GAPDH, CAATGACCCCTTCATTGACC (forward) and GACAAGCTTCCCGTTCTCAG (reverse). The primers were synthesized by Shanghai Sangon Biological Engineering Technology & Services (Shanghai, China). For real-time RT-PCR, the cDNA, primer pairs and SYBR Green dye were used in TaqMan Master Mix (Applied Biosystems). The total mix was run on a 7900 Real Time System (Applied Biosystems by Life Technologies, Carlsbad, CA, USA) with the PCR conditions as follow: 94°C (30 s), 59°C (30 s) and 72°C (45 s) for 40 cycles. The data are presented as the relative mRNA level normalized to GAPDH and then expressed as the fold increase relative to the control.

### Preparation of total cell lysates

To detect HO-1, GSK-3β and phospho-GSK-3β, total cell extracts were prepared as described, with minor modifications [26]. Cells were lysed at 4°C in a buffer containing 25 mM HEPES (pH 7.5), 0.3 M NaCl, 1.5 mM MgCl2, 0.2 mM EDTA, 0.5 mM dithiothreitol (DTT), 0.1% Triton X-100, 200 mM β-glycerolphosphate, 0.1 mM Na3VO4, 21 g/mL leupeptin and 1 mM phenylmethylsulfonyl fluoride (PMSF). The supernatants were collected, assayed for protein concentration by using the BCA (Pierce, Rockford, IL, USA) protein assay kit according to the manufacturer’s specifications, aliquoted and stored at -80°C until used for Western blot analyses.

### Preparation of cytosolic and nuclear extracts

For the preparation of nuclear extracts, the treated cells were placed on ice, the medium was removed, and the cells were washed once with cold PBS. Cells were then gently removed by mechanical scraping and collected by centrifugation at 1,500 g for 5 min. The cell pellet was resuspended in 5 mL of cell lysis buffer (10 mM HEPES (pH 7.9), 1.5 mM MgCl2, 10 mMKCl, 0.5 mMdithiothreitol (DTT), and 0.2 mMphenylmethylsulfonyl fluoride (PMSF)) and immediately centrifuged at 1,500 g for 5 min. The cells were resuspended in two-times the original packed cell volume of cell lysis buffer, allowed to swell on ice for 10 min, and homogenized with 10 strokes of a Dounce homogenizer (B pestle). Nuclei were collected by centrifugation at 3,300 g for 15 min at 4°C; the supernatant was set aside on ice for the preparation of CEs. The nuclei were resuspended, using six strokes of a Teflon-glass homogenizer, in three volumes (approximately 750 L) of nuclear extraction buffer (20 mM HEPES (pH 7.9), 1.5 mM MgCl2, 400 mMKCl, 0.5 mM DTT, 0.2 mM PMSF, and 25% glycerol). The nuclear suspension was stirred on ice for 30 min and then centrifuged at 89,000 g for 30 min. The supernatant was collected and concentrated in a Microcon 10 concentrator by centrifugation at 14,000 g for 3 h at 4°C. For preparation of cytosolic extracts, the supernatant obtained after removal of nuclei was mixed thoroughly with 0.11 volume of 10× cytoplasmic extraction buffer [1×: 30 mM HEPES (pH 7.9) at 4°C, 140 mM KCl, 3 mM MgCl2] and then centrifuged at 89,000 g for 1 h. The supernatant was collected and concentrated by centrifugation at 14,000 g for 1 h at 4°C. Protein concentrations were determined using the BCA assay (Pierce, Rockford, IL, USA).

### Western blot analysis

Equal amounts of proteins (30–50 μg) were separated by 10% sulfate-polyacrylamide gel electrophoresis (Bio-Rad Laboratories, Inc., Hercules, CA, USA) and transferred to nitrocellulose membranes (Pierce). The membranes were blocked for 2 h in a non-fat dried milk solution (5% in Tris-buffered saline) containing 0.5% Tween 20, and then incubated with appropriate primary antibodies overnight at 4°C. Specific protein expressions were revealed by enhanced chemiluminescent reagents (Thermo Scientific, Beijing, China) and detected using X-ray film. GAPDH and Histone H2 were used as loading controls for total/cytosolic extracts and nuclear extracts, respectively. The data are presented as fold-increases relative to the control.

### Transient transfection and luciferase reporter gene assay

A dual-luciferase reporter assay system (Promega, Madison, WI, USA) was used to determine promoter activity in transiently transfected cells. Briefly, L02 cells (1×10^5^cells /well) were plated in 24-well plates and cultured for 24 h. The cells were then co-transfected with 0.2 μg HO-1 promoter-encoding firefly luciferase plasmid, which was a kind gift from Professor Norbert Leitinger (University of Virginia, VA, USA) [27], and 0.02 μg pRL-TK-encoding Renilla luciferase plasmid (Promega, Madison, WI, USA) using Lipofectamine 2000 (Invitrogen). After 24 h of incubation, the cells were exposed to hyperoside or t-BHQ for the indicated times. The activities of firefly and Renilla luciferase were measured in a luminometer (BertholdTechnologies, Bad Wildbad, Germany) with the Dual-Luciferase Reporter Assay System (Promega) according to the supplier's recommendations. The luciferase activities were normalized according to transfection efficiency monitored by Renilla expression, and HO-1 transcriptional activity was expressed as a fold induction relative to that of the control cells.

### Statistical analysis

All experiments were performed at least three times, and the results were expressed as the means ± standard deviation (SD). The results were analyzed by one-way analysis of variance followed by a SNK-q test for multiple comparisons. The statistical significance of differences between two groups was determined by Student’s t-test. P-values <0.05 were considered to be statistically significant. All analyses were performed using the Statistical Package for the Social Sciences (SPSS) software (Chicago, IL, USA).

## Results

### Hyperoside-mediated cytoprotective effect was enhanced by LiCl

To explore the role of GSK-3β in the hyperoside-mediated protection against H_2_O_2_-induced oxidative damage, L02 cells pretreated with 10 mM LiCl for 30 min were incubated with 100 μM hyperoside or 50 μM t-BHQ for 24 h, and the cells were then stimulated with 100 μM H_2_O_2_ for an additional 6 h. Cell viability, LDH leakage and total GSH levels were assayed.

**Fig 2:**
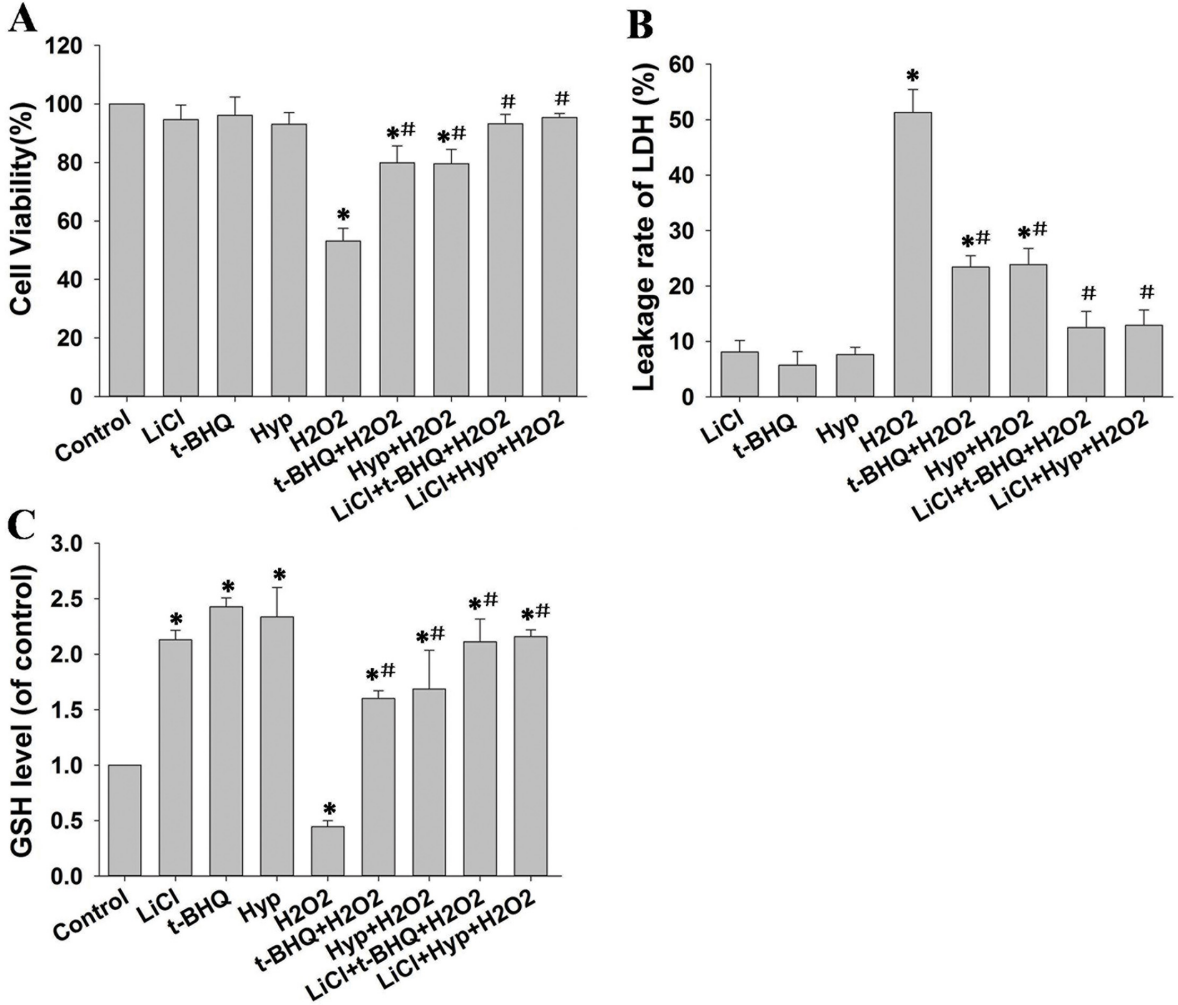
Effects of LiCl-mediated GSK-3β inhibition on hyperoside-mediated cytoprotection against H_2_O_2_-induced L02 cell damage. After pretreatment with 10 mM LiCl for 30 min, the cells were incubated with 100 μM hyperoside or 50 μM t-BHQ for 24 h and then stimulated with or without 100 μM H_2_O_2_ for a further 6 h. (A) Cell viability was measured by Cell Counting Kit-8 assay. (B) LDH levels in the culture medium were analyzed by using an LDH assay kit. (C) The levels of total glutathione (GSH) were detected with a GSH measurement kit. Data are expressed as the mean ± SD (n = 5). * p < 0.05 vs. control group. # p <0.05 vs. H_2_O_2_-only group.

As shown in **Fig 2A**, hyperoside significantly protected the L02 cells by increasing cell viability. LiCl pretreatment enhanced the cytoprotective effect of hyperoside. LiCl treatment alone had no effect on cell viability. The effect of LiCl on the capacity of hyperoside to protect cell membrane integrity was next investigated by measuring LDH levels in the culture medium following treatment with LiCl, hyperoside and H_2_O_2_. H_2_O_2_-induced cell membrane damage was markedly attenuated in the presence of hyperoside. Furthermore, the protective capacity of hyperoside was enhanced by LiCl, as shown by a cell viability assay (**Fig 2B**).

Because hyperoside increases the activity of SOD and HO-1 [6], we further examined total GSH level, another important component of the cellular defense against oxidative stress. As shown in **Fig 2C**, hyperoside or LiCl treatment alone led to significantly increased GSH levels (2.34- and 2.13-fold, respectively), compared with untreated cells. H_2_O_2_ treatment decreased the level of total GSH (to 0.45-fold of the control cell), whereas pretreatment with hyperoside alone or in combination with LiCl restored the total GSH levels (to 1.69- and 2.16-fold of the control cells, respectively). These results suggest that hyperoside counteracts oxidative damage through a mechanism involving GSK-3β inhibition.

### LiCl increased hyperoside-induced Nrf2 nuclear accumulation and HO-1 expression

**Fig 3:**
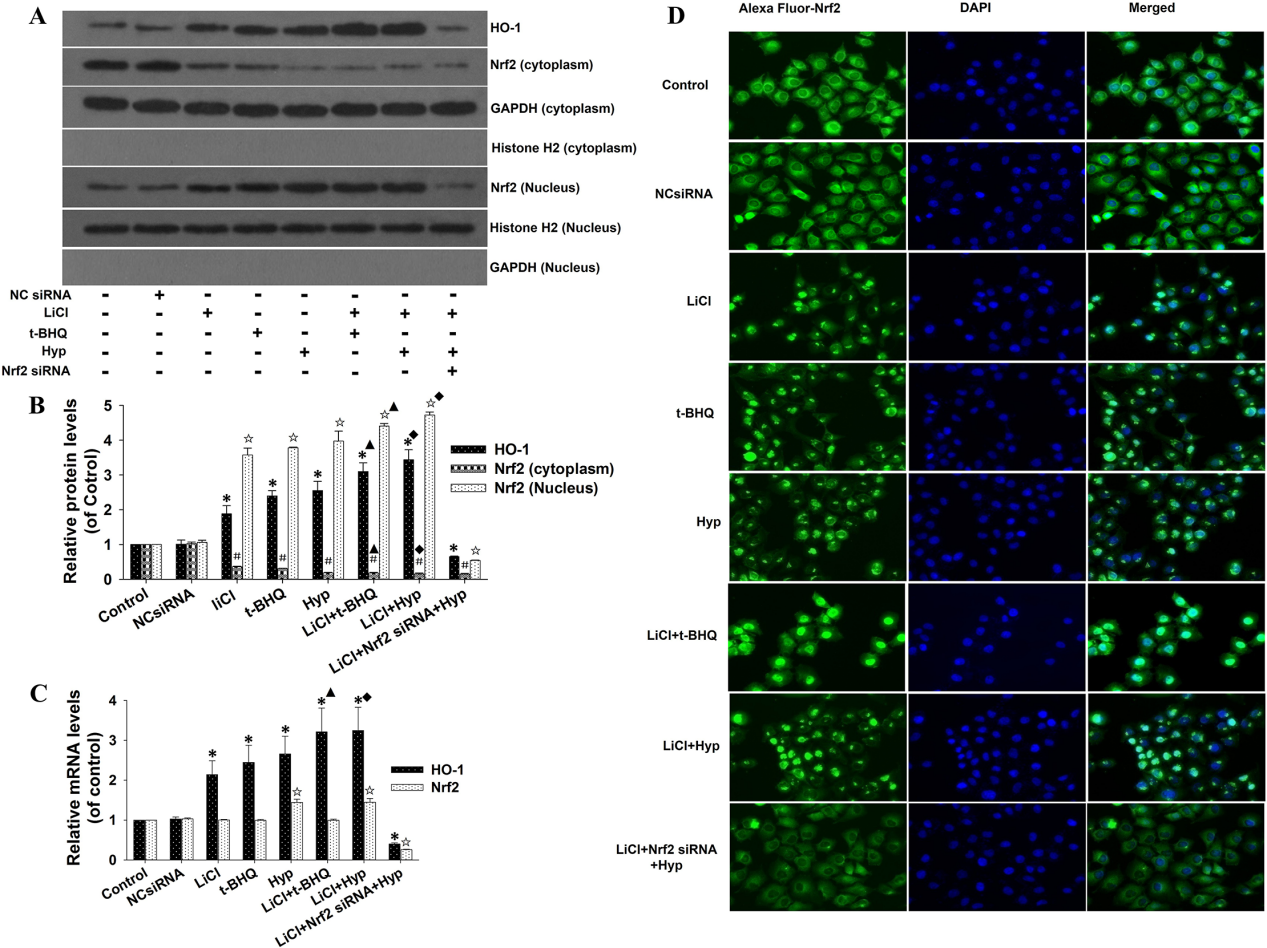
Effects of LiCl-mediated GSK-3β inhibition on the hyperoside-induced nuclear accumulation of Nrf2. After pretreatment with 10 mM LiCl for 30 min and transfection with 100 nM Nrf2 siRNA or control siRNA for 48 h, the L02 cells were incubated with 100 μM hyperoside or 50 μM t-BHQ for 6 h. (A) The total cell lysates, cytosolic extracts and nuclear extracts were prepared, and the protein expression of HO-1 and Nrf2 was examined by Western blot. GAPDH and Histone H2 were used as loading controls for cytosolic extracts and nuclear extracts, respectively. (B) Scanning densitometry was used for semi-quantitative analysis compared with control groups. (C) The mRNA expression of HO-1 and Nrf2 was analyzed by real-time RT-PCR. The measurements were made in triplicate, and the data are expressed as the mean ± SD (n = 5). *, #, ☆ p <0.05 vs. control group. ▲ p<0.05 vs. t-BHQ-only group. ■ p<0.05 vs. hyperoside-only group. (D) Fixed L02 cells were incubated with anti-Nrf2 antibody, Alexa Fluor® 488 goat anti-mouse IgG antibody and counterstained with DAPI. The nuclear translocation of Nrf2 was observed under a fluorescent microscope.

Nrf2 is a transcription factor that controls the expression of a network of antioxidant and cytoprotective genes, including *HO-1* and *GSH*, to regulate the cellular responses to oxidative and electrophilic stress. Because our previous study demonstrated that hyperoside treatment increases the expression of Nrf2 and facilitates its nuclear accumulation [6], we next investigated the role of GSK-3β inhibition by LiCl in the interaction between Nrf2 and hyperoside. We found that LiCl or hyperoside treatment alone significantly increased HO-1 protein expression and Nrf2 nuclear accumulation (**Fig 3A**). LiCl-pretreated cells exhibited higher Nrf2 protein levels in the nuclear compartment and lower Nrf2 protein level in the cytosol compartment, compared with the cells in the hyperoside-only group (**Fig 3B**). In contrast, Nrf2 siRNA inhibited Nrf2 and significantly decreased HO-1 protein expression (**Fig 3A and 3B, lane 8**).

As shown in **Fig 3C**, the mRNA levels of Nrf2 and HO-1 were significantly increased in cells treated with hyperoside alone or in combination with LiCl. LiCl treatment alone also elicited a significant increase in HO-1 mRNA but not Nrf2 mRNA. In agreement with the findings shown in **Fig 3A and 3B (lane 8**), hyperoside failed to induce HO-1 mRNA expression by Nrf2 silencing in LiCl-pretreated L02 cells.

Thus, hyperoside-induced Nrf2 nuclear accumulation and HO-1 expression was facilitated by GSK-3β inhibition. The hypothesis was supported by the immunofluorescence observations of Nrf2 nuclear translocation in response to LiCl and hyperoside exposure (**Fig 3D)**.

### LiCl enhanced hyperoside-induced HO-1 ARE-luciferase activity

**Fig 4:**
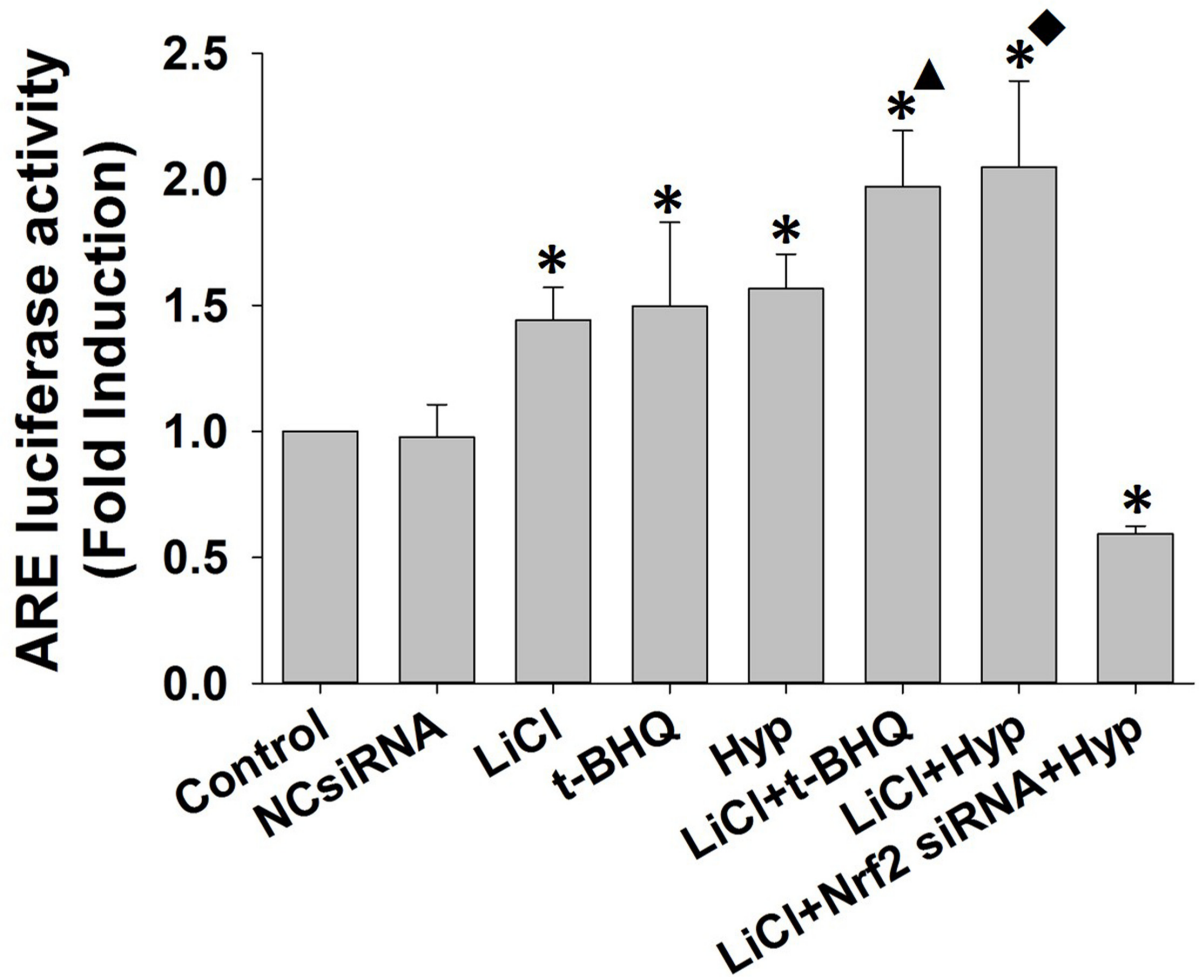
Effects of LiCl-mediated GSK-3β inhibition on the hyperoside-induced transcriptional activation of HO-1 expression. L02 cells were co-transfected with 100 nM Nrf2 siRNA, hHO4.9luc luciferase reporter plasmid and fire fly Renillaluciferase, according to the manufacturer’s instructions. After 48 h of transfection, the L02 cells were treated with 10 mM LiCl for 30 min and then incubated with 100 μM hyperoside or 50 μM t-BHQ for a further 6 h. The relative luciferase activity was examined by the Dual-Luciferase Reporter Assay System. The luciferase activity was normalized to that of untreated cells with plasmid transfection. The measurements were made in triplicate, and the data are expressed as the mean ± SD (n = 5). * p<0.05 vs. control group. ▲ p<0.05vs. t-BHQ-only group. ■ p<0.05vs. hyperoside-only group.

To ascertain whether LiCl-enhanced HO-1 expression is dependent upon ARE-mediated transcriptional activation, we performed a luciferase reporter gene assay in the cells. As shown in **Fig 4**, hyperoside and t-BHQ induced the luciferase activity. LiCl alone also caused a modest increase in the ARE reporter activation. More importantly, LiCl cooperated with both hyperoside and t-BHQ to activate HO-1 ARE reporter activation. However, hyperoside failed to increase HO-1 ARE-driven luciferase activity in cells carrying Nrf2 siRNA (**Fig 4, lane 8**). These results indicate that the inhibition of GSK-3β enzyme activity is associated with the hyperoside-activated Nrf2-ARE signaling pathway.

### Hyperoside induced GSK-3β phosphorylation in L02 cells

**Fig 5:**
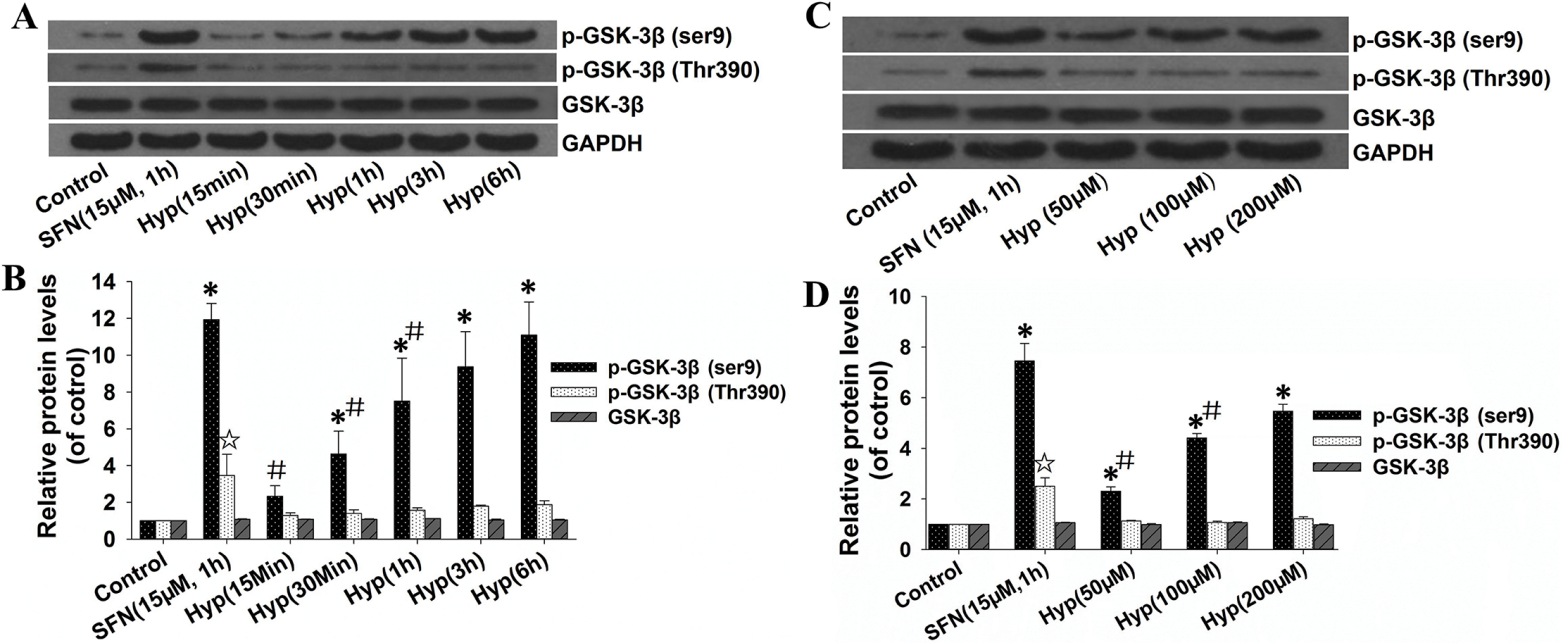
Effects of hyperoside on GSK-3β phosphorylation in L02 cells. L02 cells were cultured with 100 μM hyperoside for the indicated times (A) or increasing concentrations of hyperoside for 6 h (C). L02 cells treated with 15 μM sulforaphane for 1 h were used as a positive control. Total cellular extracts were prepared, and the protein expression of p-GSK-3β (Ser9), p-GSK-3β (Thr390) and GSK-3β was examined by Western blot. GAPDH was used as a loading control for each lane. Scanning densitometry was used for semi-quantitative analysis compared to the control groups (B and D). Each bar represents the mean ± SD from three independent experiments. *,☆ p<0.05 vs. control group. # p<0.05 vs. SFN group.

As the phosphorylation of GSK-3β at Ser9 [15] and Thr390 is known to inactivate GSK-3β [28], we tested the capacity of hyperoside to induce the phosphorylation of GSK-3β. As shown in **Fig 5A and 5B**, hyperoside-treated L02 cells exhibited increased levels of phosphorylated GSK-3β at ser9, and the level peaked at 6 h of exposure.

Hyperoside treatment also enhanced the phosphorylation of GSK-3β at Ser9 in a dose-dependent manner (**Fig 5C and 5D**). However, the protein levels of GSK-3β and phosphorylated GSK-3β at Thr390 were not significantly changed in hyperoside-treated L02 cells under the same experimental conditions (**Fig 5B and 5D**). These findings confirm that the hyperoside-activated Nrf2-ARE signaling pathway depends, at least partially, on GSK-3β activity inhibition through phosphorylation at Ser9.

### siRNA-mediated inhibition of GSK-3β enhanced hyperoside-induced activation of Nrf2-ARE pathway

**Fig 6:**
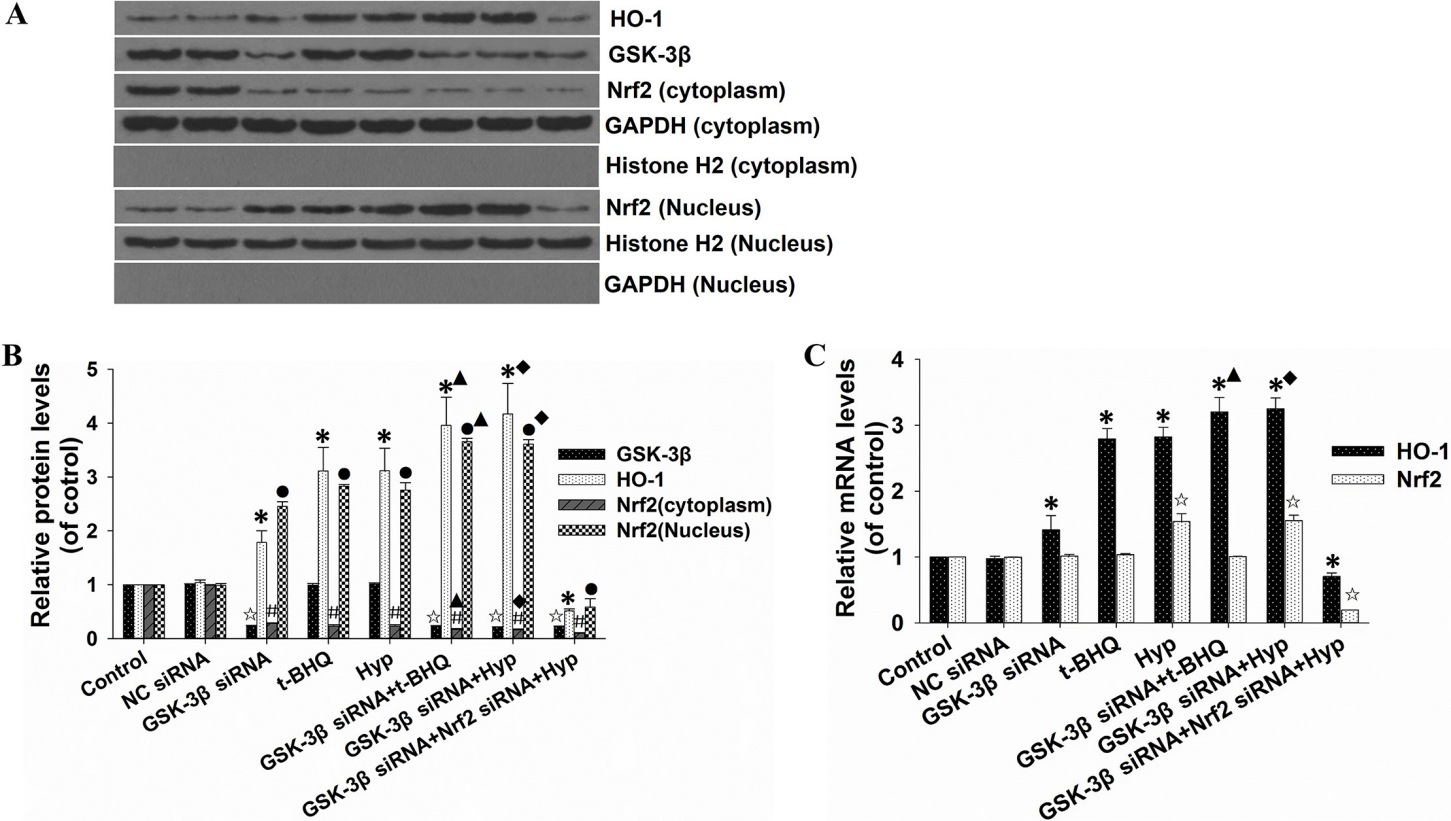
Effects of siRNA-mediated GSK-3β inhibition on hyperoside-induced nuclear accumulation of Nrf2. L02 cells were co-transfected with Nrf2 siRNA and either control siRNA or GSK-3β siRNA, according to the manufacturer’s instructions. After 48 h of transfection, L02 cells were treated with 100 μM hyperoside or 50 μM t-BHQ for 6 h. (A)The total cell lysates, the cytosolic extracts and the nuclear extracts were prepared, and the protein expression of HO-1, GSK-3β and Nrf2 was examined by Western blot. GAPDH and Histone H2 were used as loading controls for the cytosolic extracts and the nuclear extracts, respectively. (B)Scanning densitometry was used for semi-quantitative analysis, and the results were compared to the control groups. (C)The mRNA expression of HO-1 and Nrf2 was analyzed by real-time RT-PCR. The measurements were performed in triplicate, and the data are expressed as the mean ± SD (n = 3). ☆, *, #, ● p<0.05 vs. control group. ▲ p<0.05 vs. t-BHQ-only group. ■ p<0.05 vs. hyperoside-only group.

To further confirm the effect of GSK-3β suppression on the activation of the Nrf2-ARE signaling pathway by hyperoside, siRNA against GSK-3β was used. We found that siRNA-mediated inhibition of GSK-3β resulted in significantly increased nuclear accumulation of Nrf2 and protein expression of HO-1 (**Fig 6A and 6B**). The protein level of Nrf2 in the cytosol was markedly decreased by GSK-3β siRNA transfection. Moreover, silencing of GSK-3β also facilitated hyperoside-mediated induction of Nrf2 nuclear accumulation and HO-1 protein expression. However, Nrf2 nuclear accumulation and HO-1 protein expression induced by GSK-3β silencing and hyperoside were inhibited by Nrf2 siRNA (**Fig 6A and 6B, lane 8**). Interestingly, the protein expression of GSK-3β was not significantly influenced by hyperoside (**Fig 6A and 6B, lane 5**).

**Fig 7:**
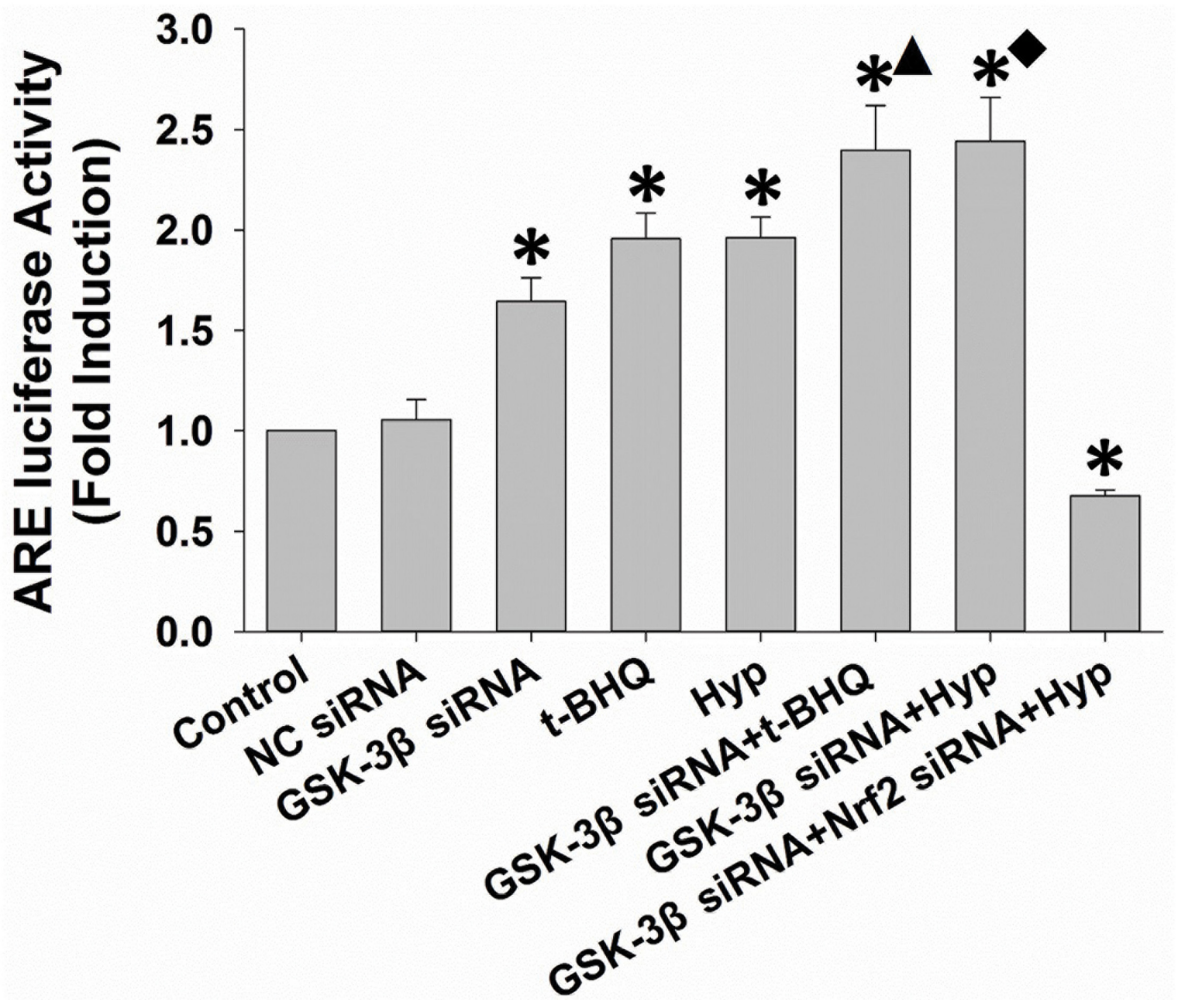
Effects of siRNA-mediated GSK-3β inhibition on the hyperoside-induced transcriptional activation of HO-1 expression. L02 cells were co-transfected with 100 nM Nrf2 siRNA, hHO4.9luc luciferase reporter plasmid, firefly Renilla luciferase and either control siRNA or GSK-3β siRNA, according tothe manufacturer’s instructions. After 48 h of transfection, the L02 cells were treated with 100 μM hyperoside or 50 μM t-BHQ for 6 h. The relative luciferase activity was examined with the Dual-Luciferase Reporter Assay System. The luciferase activity was normalized to that of untreated cells with plasmid transfection. The measurements were performed in triplicate, and the data are expressed as the mean ± SD (n = 5). * p<0.05 vs. control group. ▲ p<0.05 vs. t-BHQ-only group. ■ p<0.05 vs. hyperoside-only group.

As shown in **Fig 6C**, the mRNA level of *HO-1* was significantly increased in cells treated with hyperoside, GSK-3β siRNA or their combination. Interestingly, a marked increase in the Nrf2 mRNA level was observed only in hyperoside-treated, but not in GSK-3β siRNA-transfected cells. L02 cells transfected with Nrf2 siRNA exhibited a similar decrease in mRNA levels of Nrf2 and HO-1 as their protein expressions (**Fig 6A and 6B, lane 8**). In agrement with the results shown in **Fig 4**, HO-1 ARE-luciferase activity was increased in cells transfected with GSK-3β siRNA, treated with hyperoside or both. In contrast, the luciferase activity was significantly decreased in the presence Nrf2 siRNA (**Fig 7**). These results indicate that the inhibition of GSK-3β at the post-translation levels is involved in hyperoside-mediated the Nrf2-ARE signaling pathway induction.

### siRNA-mediated inhibition of Keap1 provided an additive effect with GSK-3β siRNA on the enhancement of hyperoside-induced activation of the Nrf2-ARE pathway

**Fig 8:**
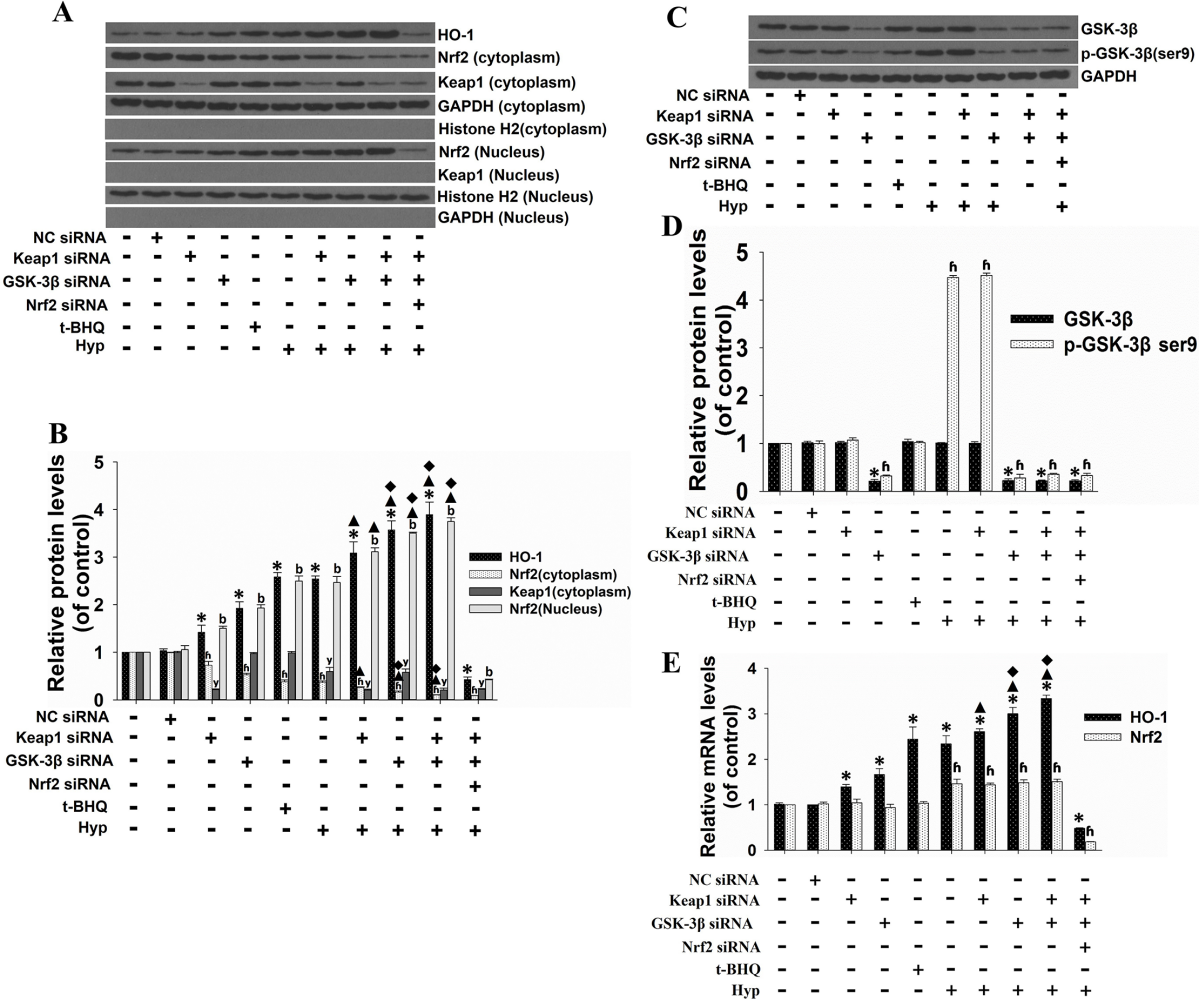
Effects of siRNA-mediated Keap1 inhibition on the hyperoside-induced Nrf2 nuclear accumulation and GSK-3β phosphorylation in L02 cells. L02 cells were co-transfected with Keap1 siRNA and either control siRNA or GSK-3β siRNA, according to the manufacturer’s instructions. After 48 h of transfection, the L02 cells were treated with 100 μM hyperoside or 50 μM t-BHQ for 6 h. The total cell lysates, the cytosolic extracts and the nuclear extracts were prepared, and the protein expression of HO-1, Nrf2, Keap1, GSK-3β and p-GSK-3β (Ser9) was examined by Western blot. GAPDH and Histone H2 were used as the loading controls for cytosolic extracts and nuclear extracts, respectively (A and C). Scanning densitometry was used for the semi-quantitative analysis compared to the control groups (B and D). Each bar represents the mean ± SD from three independent experiments. *, ʱ, ʸ, b p<0.05 vs. control group. ▲p<0.05 vs. hyperoside-only group. ◆ p<0.05 vs. Hyp+Keap1 siRNA group. (E)The mRNA expression of HO-1 and Nrf2 was analyzed by real-time RT-PCR. The measurements were performed in triplicate, and the data are expressed as the mean ± SD (n = 3). *, ʱ p<0.05 vs. control. ▲p<0.05 vs. hyperoside-only group. ◆ p<0.05 vs. Hyp+Keap1 siRNA group.

It is known that Keap1 is another inhibitor of Nrf2, and our previous data has indicated that hyperoside induces Keap1 inhibition probably by facilitating Keap1 protein degradation [6]. To provide insight into how significantly hyperoside-mediated GSK-3β inhibition contributes to the Nrf2-ARE signaling pathway induction, siRNA against Keap1 was used. Keap1 siRNA alone resulted in a significantly increased nuclear accumulation of Nrf2 and protein expression of HO-1 (**Fig 8A and 8B**). The protein level of Nrf2 in the cytosol was markedly decreased by Keap1 siRNA transfection. Moreover, siRNA-mediated inhibition of Keap1 also facilitated hyperoside-mediated induction of Nrf2 nuclear accumulation and HO-1 protein expression. However, Nrf2 nuclear accumulation and HO-1 protein expression induced by Keap1 silencing, GSK-3β inhibition and hyperoside were inhibited by Nrf2 siRNA. Most interestingly, the enhanced effects on hyperoside-induced activation of the Nrf2-ARE pathway mediated by GSK-3β inhibition were more significant than those obtained from Keap1 silencing (**Fig 8A and 8B, lanes 7 and 8**). As shown in **Fig 8E**, the mRNA level of *HO-1* was significantly increased in cells treated with hyperoside, Keap1 siRNA, GSK-3β siRNA or their combination. Moreover, Keap1 siRNA administration alone had no significant effects on the phosphorylation and protein expression of GSK-3β or on the mRNA level of *Nrf2* (**Fig 8C, 8D and 8E, lane 3**). Conversely, GSK-3β silencing also had no significant influence on Keap1 protein levels in the cytosol (**Fig 8A and 8B, lane 4**). In agreement with our published data, Keap1 was not detected in the nucleus under the same experimental conditions. These results suggest that hyperoside activates the Nrf2-ARE signaling pathway both grossly by inhibiting GSK-3β at the post-translation levels and slightly by decreasing the Keap1 protein level.

## Discussion

Due to the predominant role of hepatocytes in the biotransformation and metabolism of xenobiotics, ROS production constitutes a severe burden in liver pathophysiology and hence in the progression of liver diseases [29, 30]. It is widely believed that natural antioxidant compounds might pave a way for the therapy of liver diseases [31, 32]. Hyperoside, a major active constituent in many medicinal plants, is traditionally used for its anti-inflammatory and antioxidative effects. In this study, we confirmed that hyperoside protects L02 cells against H_2_O_2_-induced damage via an Nrf2-ARE signaling pathway. We also demonstrated that the inactivation of GSK-3β is crucial for hyperoside-mediated Nrf2 nuclear accumulation and HO-1 expression.

Our previous study suggested that ERK and p38 signaling pathways participate in hyperoside-mediated Nrf2 nuclear translocation. However, other pathway(s), such as PKC and PI3K, are also believed to be involved [6]. GSK-3β is tightly regulated by the survival pathway represented by PI3K/Akt and other kinases including ERK and PKC [15, 28]. We therefore investigated the effect of hyperoside on the expression and phosphorylation of GSK-3β. We showed that hyperoside was capable of inducing the phosphorylation of GSK-3β at Ser9, consistent with previous findings which indicate that the plant antioxidant compounds sulforaphane, quercetin and puerarin enhance the inhibitory Ser9-phosphorylation of GSK-3β and Nrf2 nuclear translocation in diabetic nephropathy models, normal bronchial epithelial cells and neuronal cultures [18, 33, 34]. Interestingly, the protein levels of GSK-3β and the phosphorylation of GSK-3β at Thr390 were not significantly influenced by hyperoside. These findings are in agreement with a recent study showing that the flavonoid quercetin regulates Nrf2 through the PI3K/AKT pathway without affecting the gene expression of *GSK-3β* in mice [19]. Our results, however, are inconsistent with those of a previous study, in which nordihydroguaiaretic acid was found to cause inhibitory phosphorylation of GSK-3β at both Ser9 and Thr390, and this was associated with a substantial increase in the Nrf2 stability in mouse embryo fibroblasts [15]. In addition, p38 MAPK-mediated inhibitory phosphorylation of GSK-3β at Thr390 occurred primarily in the brain and thymocytes [28]. However, hyperoside failed to induce the phosphorylation of GSK-3β at Thr390 in our experiments, even though the p38 signaling pathway has been suggested to participate in hyperoside-mediated Nrf2 activation. These discrepancies might be because GSK-3β can be differentially regulated under diverse experimental conditions.

Because an increasing body of literature has indicated GSK-3β as a negative regulator of Nrf2 [13], we investigated the role of the chemical-based GSK-3β inhibitor, lithium chloride, in Nrf2 activation and HO-1 induction by hyperoside. We demonstrated that LiCl significantly enhanced the ability of hyperoside to protect L02 liver cells from H_2_O_2_-induced oxidative damage and increased the GSH level, one of the endogenous antioxidant biomarkers. These results suggested that the inhibition of GSK-3β activity participates in hyperoside-mediated cytoprotective effects and up-regulation of the intracellular antioxidant enzyme system.

HO-1, one of the most readily induced antioxidant enzymes under oxidative stress conditions [35], decomposes hemin, a toxic oxidant, to bilirubin, carbon monoxide and ferrous efflux, thereby providing cell protection against oxidative stress [36]. In this study, LiCl pretreatment significantly enhanced hyperoside-induced HO-1 expression, which was dependent upon Nrf2 nuclear translocation and gene expression followed by ARE-mediated transcriptional activation in the presence of hyperoside. These effects of LiCl were abolished by transfection with a specific siRNA of Nrf2, confirming the role of the GSK-3β in the activation of Nrf2 mediated by hyperoside. A particularly interesting finding in this study was that LiCl treatment alone induced the nuclear accumulation of Nrf2 but did not influence Nrf2 expression, indicating that LiCl-mediated GSK-3β inhibition regulates Nrf2 at post-translational levels in response to hyperoside. Jiang Y et al. have recently reported that chronic hepatitis C patients who received long-term lithium carbonate therapy primarily for concomitant psychiatric disorders exhibit significantly less liver injury [14], providing an interesting opportunity for the combination of LiCl and hyperoside or other Nrf2 inducers to reduce the adverse effects of LiCl.

The more significant enhanced effects of GSK-3β inhibition on hyperoside-induced Nrf2 nuclear accumulation and HO-1 protein expression compared with those obtained from Keap1 silencing and the lack of obvious change in the protein level of GSK-3β and Keap1 in the siRNA-mediated inhibition of each other, indicated that the Keap1-dependent and GSK-3β-dependent mechanisms activate the Nrf2-ARE signaling pathway in isolation, and GSK-3β is probably a major regulator of these signaling pathways. Rojo AI et al. have suggested that several kinase cascades (p38, JNK and ERK1/2) might combine to modulate Nrf2, and such signaling integration might take place partially at the level of GSK-3β [15]. A previous work from our group suggested that phosphorylation of ERK, p38 and other yet undefined signaling kinase(s), such as PKC and PI3K, participate in the hyperoside-mediated Nrf2 nuclear translocation [6]. We speculate that GSK-3β acts as an integrator of these kinases in the hyperoside-mediated Nrf2-ARE signaling pathway activation.

The active form of GSK-3β phosphorylates Fyn at threonine residue(s), which causes nuclear accumulation of Fyn and subsequent phosphorylation of Nrf2 at tyrosine 568, followed by the nuclear export, ubiquitination, and degradation of Nrf2 [13, 37, 38]. GSK-3β also phosphorylates a group of Ser residues in the Neh6 domain of mouse Nrf2, which leads to SCF/beta-TrCP-dependent degradation of Nrf2 [12, 39, 40]. It is possible that the inhibition of GSK-3β activity or a decrease in the protein level of active GSK-3β induced by hyperoside through phosphorylating GSK-3β at Ser9 leads to inhibition of Fyn-mediated Nrf2 nuclear export or GSK-3β-induced Nrf2 phosphorylation in the Neh6 domain. Although the causal relationship remains unclear, the enhancement of inhibitory Ser9-phosphorylation of GSK-3β and Fyn/Nrf2 nuclear export/import by SFN helps chart course for further study.

Interestingly, we also found that hyperoside, either with or without GSK-3β siRNA and/or Keap1 siRNA, enhanced *Nrf2* expression at the mRNA level, indicating that transcriptional and/or post-transcriptional mechanisms control the expression of this transcription factor independent of GSK-3β and Keap1. MicroRNAs are normally considered as fine-tuning regulators of protein production through post-transcriptional alterations and/or translation repression. Narasimhan M et al. obtained evidence that the Nrf2 mRNA abundance and nucleo-cytoplasmic concentration of Nrf2 could be directly regulated by miR153/miR27a/miR142-5p/miR144 in a Keap1-independent manner in SH-SY5Y dopaminergic neurons [41, 42]. Recently, a report from another group confirmed that miR27b regulated the Nrf2 mRNA level by binding to its 3’-UTR region in the liver and hepatocytes, resulting in a decreased level of Nrf2 protein and expression of Nrf2-dependent antioxidant genes[43]. Therefore, we speculate that the expression of Nrf2 might be post-transcriptionally regulated by microRNA(s) in hyperoside-treated L02 cells, which lead to an increase of Nrf2 mRNA levels. Moreover, because Nrf2 is itself positively regulated by ARE at the promoter level [44], the finding of higher Nrf2 mRNA levels with hyperoside-treatment provides clues that some transcription co-activators of Nrf2 might be induced by hyperoside to activate ARE.

**Fig 9:**
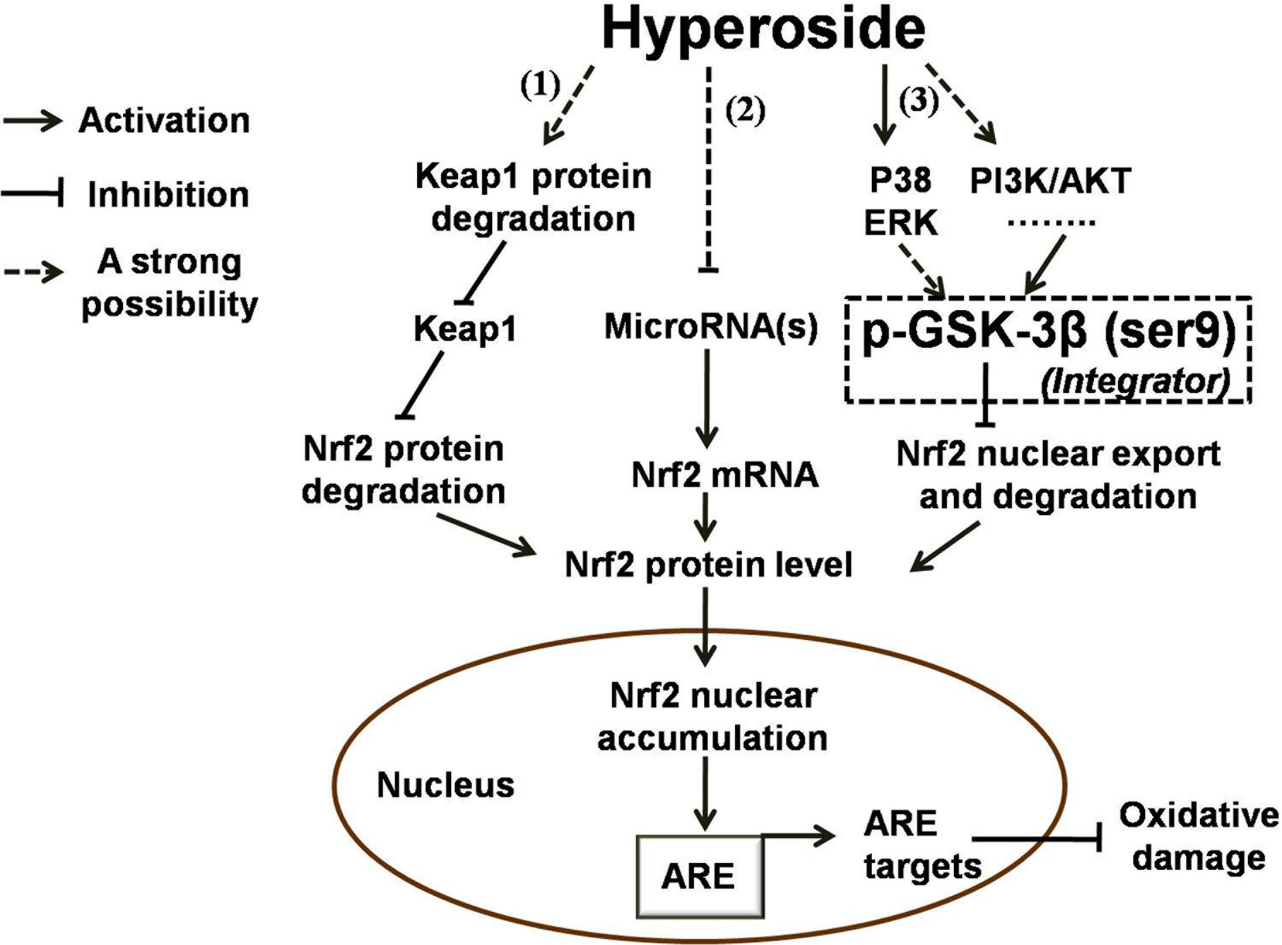
Schematic overview of regulations in hyperoside-mediated Keap1-Nrf2-ARE signaling pathway activation. (1) Hyperoside decreases the Keap1 protein level, most likely by facilitating Keap1 protein degradation, which resulted in a drop in Nrf2 protein degradation; (2) MicroRNA(s), which has been proposed to target Nrf2 mRNA by binding to its 3′-UTR and result in decreased levels of Nrf2 protein, might be involved in hyperoside-mediated increases in the Nrf2 mRNA level; (3) GSK-3β possibly acts as an integrator of a number of kinases such as ERK, p38 and other yet undefined signaling kinase(s) in hyperoside-mediated Nrf2 activation. All of the above mentionedmechanisms lead to increases in Nrf2 nuclear accumulation and Nrf2 target gene expression. (The dashed line highlights that this is a strong suggestion and has not been experimentally proven).

In conclusion, our work provides the first evidence that hyperoside attenuates H_2_O_2_-induced L02 cell damage by activating the Nrf2-ARE signaling pathway via GSK-3β inactivation. A summary of our findings is shown in **Fig 9**. Future research will explore the mechanisms underlying the regulatory effect of hyperoside on GSK-3β and the cross-talk among GSK-3β, the PI3K/AKT pathway, microRNAs and other pathways, for a better understanding of the therapeutic potential of hyperoside for oxidative liver disorders.
